# An investigation of privacy preservation in deep learning-based eye-tracking

**DOI:** 10.1186/s12938-022-01035-1

**Published:** 2022-09-13

**Authors:** Salman Seyedi, Zifan Jiang, Allan Levey, Gari D. Clifford

**Affiliations:** 1 Biomedical Informatics, School of Medicine, Emory, Atlanta, USA; 2 Biomedical Engineering, Georgia Institute of Technology, Atlanta, USA; 3 Neurology, School of Medicine, Emory, Atlanta, USA

**Keywords:** Data leakage, Facial features, Eye-tracking, Deep neural networks

## Abstract

**Background:**

The expanding usage of complex machine learning methods such as deep learning has led to an explosion in human activity recognition, particularly applied to health. However, complex models which handle private and sometimes protected data, raise concerns about the potential leak of identifiable data. In this work, we focus on the case of a deep network model trained on images of individual faces.

**Materials and methods:**

A previously published deep learning model, trained to estimate the gaze from full-face image sequences was stress tested for personal information leakage by a white box inference attack. Full-face video recordings taken from 493 individuals undergoing an eye-tracking- based evaluation of neurological function were used. Outputs, gradients, intermediate layer outputs, loss, and labels were used as inputs for a deep network with an added support vector machine emission layer to recognize membership in the training data.

**Results:**

The inference attack method and associated mathematical analysis indicate that there is a low likelihood of unintended memorization of facial features in the deep learning model.

**Conclusions:**

In this study, it is showed that the named model preserves the integrity of training data with reasonable confidence. The same process can be implemented in similar conditions for different models.

## Introduction

The importance of exploring guidelines and regulations regarding implementation for different machine learning (ML) and artificial intelligence (AI) techniques increases as these techniques become prevalent in various settings that involve private individual data. In the US, in the context of health-related data and protected health information (PHI), the Health Insurance Portability and Accountability Act (HIPAA) of 1996 defines how information must be scrubbed prior to use outside of a protected enclave. HIPAA’s primary goals are providing regulation to facilitate the portability of the data and preventing leakage of PHI. It has been shown that sharing seemingly benign healthcare data can result in unintended PHI leaks; for instance, the electroencephalogram (EEG) [1] or electrocardiogram (ECG) [2] or even data from sensors of the wearables can be used to re-identify the participant [3], as long as the pool of people from whom you sample is relatively small (rendering the approach relatively useless in practice). Another example of the potential leak of private data comes from wearable cameras, and one attempt to address it is by Stein et al. [4]. They approach the concerns about a camera recording sensitive/personal situations in daily use of an augmented reality wearable camera by implementing AI to control a physical shutter to block the camera when appropriate.

While these are all examples of raw data (or model raw output) potential for containing PHI, there are other vulnerabilities to be mindful of. Indeed one of the main concerns in the increased use of deep learning models in the different private data or PHI is that these models, with an extensive number of variables and parameters, have the potential of encoding personal details [5] and, when shared, can result in an unintended data leak [6]. These vulnerabilities seem to be exploitable not only by black box attacks [7] using only the outputs of models, but through the calculation of the gradients, loss, and other derivable parameters of the model and different inputs [8, 9].

To mitigate the problem of an information leak in deep learning or other machine learning models, differential privacy (DP) has a mathematically robust foundation to calculate and manage the privacy costs in the training of a model [10, 11]. While very impressive, DP has several shortcomings that prevents it from being utilized in all machine learning and deep learning model training. One of the issues is the difficulties in the proper implementation of the DP in models. While there has been a significant effort to ease the implementation of DP in different platforms, such as the TensorFlow privacy or Opacus library for PyTorch, incorrect implementation or incorrect privacy cost calculations can lead to a false sense of security [12] which can be very dangerous (even for large multinational corporations [13]). Moreover, the performance of the application models can suffer drastically when DP is used, especially when the size of the training set is limited [14, 15]. While there have been efforts to balance between the model performance and the mathematical guarantee of the preservation of privacy [11], there are many applications that are very sensitive to the accuracy of their model, where even a slight drop in the performance of the model can render the whole model obsolete (including the model investigated in this work [16]). Maybe even more disturbing is that this impact is enhanced in under-represented and marginalized groups and enhances the unfairness of the models [17], even with small data imbalances and loose privacy guarantees [18]. The issue of fairness is a critical concern in healthcare in general and machine learning approaches in healthcare in particular. We are obligated both ethically and in terms of the requirements of funding institutions to be cognizant of these biases. There are known biases (such as the color of the skins of participants) present in our cohort too, and even disregarding the potential unknown biases, any practice that exacerbates those biases is undesirable. Adding the model’s sensitivity to the accuracy of the gaze estimations, DP would not be suitable for our case.

**Fig. 1:**
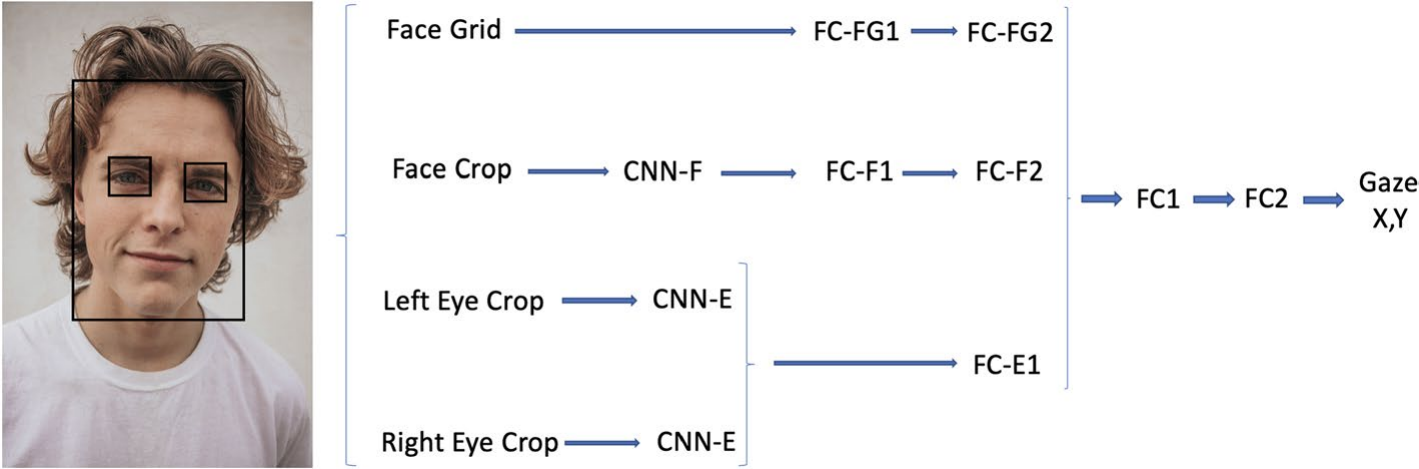
Eye gaze: illustration of the main part of the target model, which is the focus of the attack: FCs refer to different fully connected layers, while CNNs are convolutional neural network parts. After face and eye detection with regression tree, the left-eye and the right-eye are fed into CNN-E, which is CNN for eyes (shared weights) and a separate CNN, where face crop is the input (CNN-F). The photograph of the face is a modified from a publicly available image [28] under the Unsplash License [29]

Convolutional neural networks (CNN) can be particularly complex. The increased adoption of CNNs in the context of facial analysis and medical imaging [19, 20] raises concerns over their ability to encode private data. This work, therefore, explores a CNN-based model to stress-test under inference attacks, developed for an eye-tracking task [21], designed to estimate the severity of illness in cognitively impaired individuals [21]. It has been shown that eye-gaze activity data can be used to infer insights on many other medical conditions, where personal information security is paramount, such as the diagnosis of or autism disorder [22]. Moreover, researchers have been developing privacy-preserving methods to address concerns about the encoding of identities in eye-gaze data [23–27]. In this work we address whether a specific deep neural network used for eye-tracking [21] encodes information about individual identities, in addition to the eye-gaze coordinates generated by the network. This eye-tracking model can be divided into three parts. The first part involves a regression tree for face and eye detection. This detects the face and eyes from each frame in a recording. The second part, which is CNN-based and is the core of the pipeline, consists of three CNNs, one for each eye and one for the face, followed by a fully connected (FC) neural network for eyes, face, and face grid. Then, the outputs of three fully connected network come as inputs to another fully connected network to estimate the eye gaze relative to the camera position. These parts are illustrated in more detail in Fig. 1. The third part involves a support vector regression over each recording to enhance the accuracy in the eye-tracking model, but is not included in this study, since it compresses inputs into two numbers (coordinates on a screen) and has little potential for encoding individual information. The main potential vulnerability lies in the CNN component of the system, where the face and eyes are processed by a large number of weights, and could, therefore, have the potential to memorize the facial features of the participants. More details of the target model can be found in Haque et al. [21].

The key contributions in this paper are (1) The formulation of the privacy attack model and (2) the demonstration that the algorithm that analyzes aspects of the human face is not specific to any individual (at least with the complexity observed in our real-world model) and is unlikely to leak PHI. The order in which this article is presented is as follows; First, the results of the research are presented in “Results” section. “Discussion” section includes discussions on the interpretation of the results. In “Conclusion” section, the summary and conclusion can be found. In “Materials and methods” section, all materials and methods are presented. For a deep and critical understanding of the work, one might find it more helpful to jump to “Materials and methods” section before continuing from the result section.

## Results

**Fig. 2:**
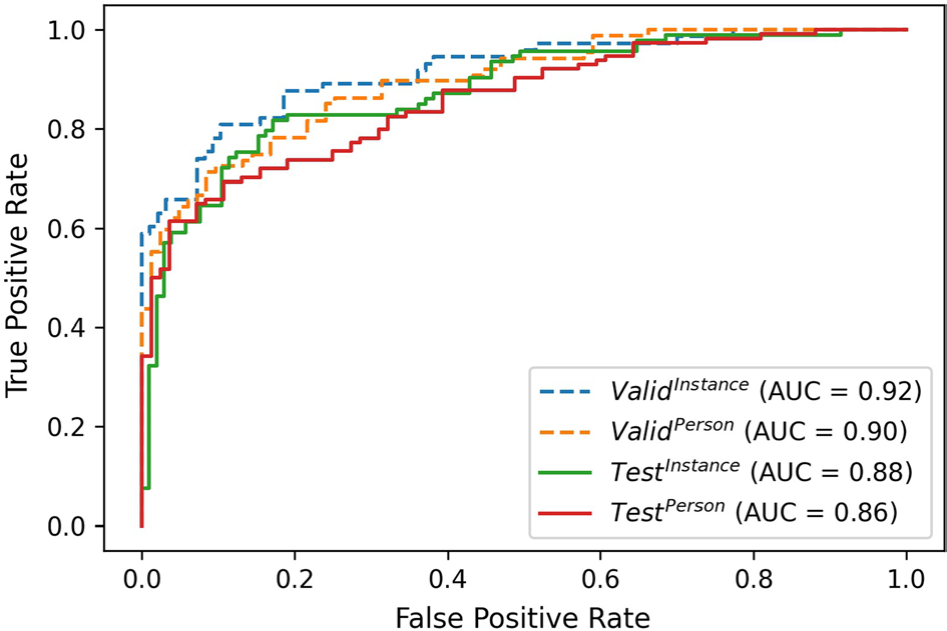
ROC curve (SVM on labeling video recordings): the dash-lines correspond to the validation set, while the solid lines are for the test set. The area under the curve for all sets and labels has been shown in the legend. While the blue and green are for the data set with instance labeling, the orange and red indicate values for the data set with person labeling

**Fig. 3:**
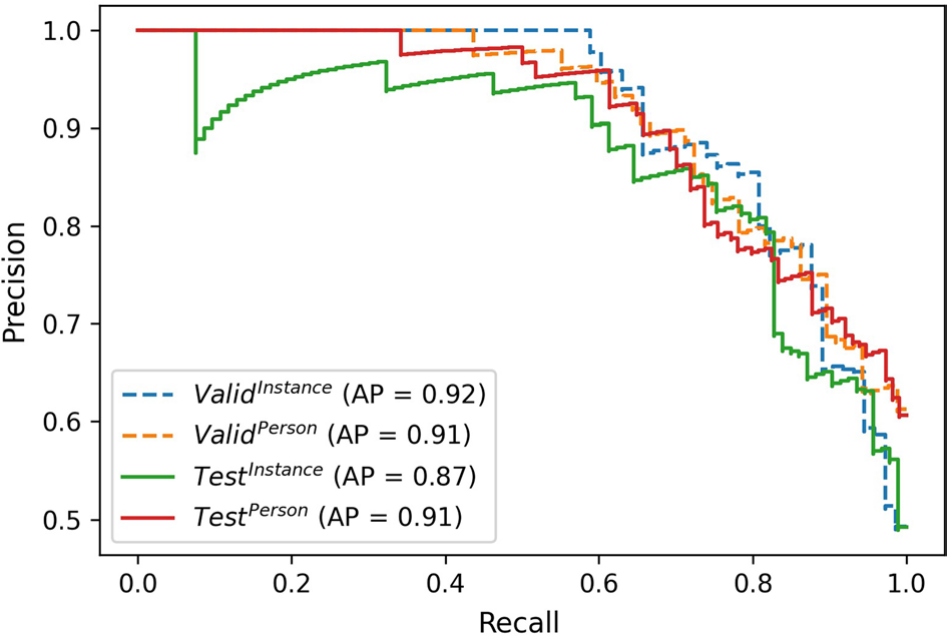
PR curve (SVM on labeling video recordings): the dash lines correspond to the validation set, while the solid lines are for the test set. Average precision scores are also provided in the legend as AP. While the blue and green are for the data set with instance labeling, the orange and red indicate values for the data set with person labeling

For the membership inference step of the pipeline, (“Record membership inference”) the performance of the support vector machine (SVM) is illustrated in Figs. 2 and 3, where the receiver operating characteristic (ROC) curve and precision–recall (PR) curve and trapezoidal area under the receiver operating characteristic curve (AUROC) and average precision (AP) have been shown for both validation and test sets with instance and person labels. The accuracy, F1-score, AUROC and AP are shown in Table 2.

Table 3 summarises the performance when considering only the records, where the instance and person labels are different.

## Discussion

The three-stage pipeline was designed to attack the target model in order to investigate the potential memorization of participants’ facial information within the model weights. One should note that the labels of the target model are the gaze coordinates of participants. It is far more challenging to harvest information from such a model, compared to a typical classification model, (for example the model used in Ref. [8]), because the facial properties of a person are not correlated with the labels in a general sense. For instance, two people with very different facial features can look at exactly the same place in an image. In addition, it is worth noting that there are other attack models for other types of target models, for example, generative models [30–32]. For instance, Chen et al. [32] propose a novel and widely applicable method for membership inference attack and privacy analysis in Generative models. However, since the target model of this study is not generative, we do not emphasize those works.

The pipeline design was adopted, because the target model is not a simple network and there are different lengths of frames for recording. In addition, this design reduces the number of learning parameters of the membership inference model. Therefore, the record membership inference and frame classifier were not trained together in an end-to-end manner. Table 1 shows that the use of only outputs of the target model for the frame labeling part of the attack confers no advantage over a random classifier. This indicates that a simple black-box attack that only uses outputs is likely to fail.

**Table 1:** Validation loss (binary cross-entropy) scores for different inputs for the frame labeling network (instance, person)

	2 output^a^	+2 grad^b^	+5 grad^c^	+2 grad + loss + label^d^	+3 grad + loss + label^e^
loss_Instance_	0.7	0.61	0.61	0.57	0.57
loss_Person_	0.7	0.61	0.62	0.59	0.58

For reference, the baseline (binary cross-entropy of random guesses for a balanced data) would be $- \log(1/2) = 0.693$.

^a^Takes the two last outputs (the target model output and the layer just before it) of the target model as the input

^b^Takes the two last outputs and also the two gradients before the last gradient of the target model

^c^Takes all the “+2 grad” and also the last gradient of three different sections of the target model (boundary, face, eyes)

^d^Takes the two last layers outputs and also the two before the last gradient and label and loss of target model

^e^Takes the two last layers outputs and also the three last gradients and label and loss of target model

As seen from Table 1, adding different gradients and also labels and loss of the target model make the classification network much more useful to decipher the correct labels both for the person and instance labels/models. The model with the highest performance on the validation set was selected for this part of the pipeline. The labels for each frame were taken as the input for the next step, where a linear SVM was selected as it gave the highest values for AP and AUROC. The values in Table 2 and also Figs. 2 and 3 show the performance of this third part of the pipeline. These results demonstrate that the labeling was reasonably successful in determining if a recording has been part of the target model training set or not. This success may be illustrated even more when compared to the accuracy of attacks with values ranging from 58.4 to 75.4% listed in table IX from Nasr et al. [8].

**Table 2:** Performance metrics for SVM in validation and test data with both instance and person label

	Valid_instance_	Valid_person_	Test_instance_	Test_person_
Accuracy	0.85	0.81	0.79	0.77
F1-score	0.82	0.80	0.80	0.79
AUROC	0.92	0.90	0.88	0.86
AP (average precision)	0.92	0.91	0.87	0.91

Threshold have been adjusted to achieve the best performance on the validation set in each model (0.68 for instance and 0.8 for person model).

One may, therefore, deduce that some part of the participants’ information is recorded in the trained network and can be extracted successfully to identify them. However, this does not translate to an identifiable information leak. First, for this attack, the assumption is that the attackers not only have full access to the target model but also access to a third of the recordings with the knowledge that they have been in the training set of the target model. Moreover, they have access to the other two-thirds of the training recordings, although they are mixed with a similar number of recordings which were not in the training set. The attacker, therefore, only has to infer which half of those belong to the training set. However, the full face cannot be recovered, and therefore, the attacker has no new information over and above membership in the training set.

The results in Table 3 support the above assertion. While for the training set 16 out of 19 recordings are identified, which in terms of significance produce the *P* value = 0.004, the validation and test set performance drops to 8 out of 14 and 12 out of 21, which is close to random. This suggests that the high performance of the pipeline in differentiating between the instances or people in and out of target training set does not come from the facial features of the participants but other aspects and features of the specific frames in the set. One limitation of this conclusion is that the number of training samples here is much smaller than the number of recordings of people with single appearance in the data set and the conclusion can potentially be changed with more recordings from each individual. However, the argument that more data can change the results can always be raised in any specific data-dependent analysis.

**Table 3:** Performance for people with multiple recordings

	Train	Valid	Test
Total^a^	19	14	21
${{\text{Instance}}^{\text{model}}}^{\text {b}}$	11	4	11
${{\text{Person}}^{\text{model}}}^{\text {c}}$	16	8	12
1 − *P*-value^Person^	0.996	0.21	0.44

^a^The total number of recordings in each set that belongs to a person who has another recording present in the training set of the target model (eye-tracking model)

^b^Only provided for the sake of completeness and is the number of picked recordings as inside (predicted label 1), despite being trained on them with labeling as outside (label 0)

^c^The number of these recordings that had been picked as inside (predicted label 1) in model trained on person labels

While techniques such as differential privacy (DP) can guarantee mathematically provable privacy preservation and robustness against many attacks [10], as discussed in “Introduction” section, it has other limitations for implementing in cases such as our target model, especially the drop in accuracy and bias against underrepresented communities. In practice, the availability and portability of the data are also critical. While one needs to take all the measures to protect sensitive or private data, it is also essential to be aware that no golden bullet is present to implement in every context.

## Conclusions

While the proposed pipeline exhibits good performance for differentiating between recordings in and out of target model training, an analysis with multiple recordings captured from given individuals demonstrates that the performance of a classifier drops to the level of a random guess when attempting to identify whether an individual appeared in the training set. This provides strong evidence that it is unlikely that recognizable facial features are recorded in the target model. In conclusion, the key contribution to the field in this work is the demonstration that it is possible to process facial characteristics that are related to behavior and health without encoding individual-specific behavior or information. While this does not preclude future successful attacks that may reveal information about an individual, the results indicate this is unlikely with current technology. It is important to note that Nasr et al. [8], who took a similar attack approach as the one presented here, found that the models they attacked did seem to encode identities, while we found the opposite. This is because the success or failure of an attack is a function of the model structure and data composition, as well as the attack itself. The purpose of this study was to ask this question about the specific model we are using, and the population we are studying. In this specific case, we demonstrated that this combination of a model and data did not create a significant risk of privacy leakage, and therefore, we are confident the presented model can be used in a clinical environment without significant risk or exposing the identity individuals used to develop the algorithm, or any user undergoing testing with the framework. One limitation of this work is that it cannot exhaustively prove this is true for all data and all models, and as such, any new training data or change in model architecture would require the reassessment of the risk using a framework such as the one presented in this article.

## Materials and methods

### Data set

The data set used in this work contains 610 video recordings from 493 participants in the Emory Healthy Aging Study undergoing an eye-tracking-based evaluation of neurological function and are described in Haque et al. [16, 21] and Jiang et al. [20]. The videos are recorded in 30 frames per second and are closeups of participants. IPad Air 9.7 inch tablets with screen 154 × 203 mm (resolution 1536 × 2048 pixels) and camera resolution 720 p were used [21]. The error rate at this section is about 3.9 (cm) [21]. Each video is 4–5 min long.

### Methodology

The primary approach of this study in investigating the potential memorization of facial information in an eye-tracking model [21, 33] (herein referred to as the “target model”) is in two general aspects. The first aspect is the analysis of pipeline performance over the membership inference of recordings. To evaluate the performance, well-known statistical tools and metrics such as receiver operating characteristic (ROC curve and PR curve and AUROC and AP would be implemented and measured. In this approach, the overall success of the attacking pipeline in differentiating between recordings used in the target model training and the recordings that were not would be a metric for the amount of data memorization in the target model.

The second aspect is to inspect if the recorded data in the model and investigated in the first aspect is related to the facial futures of the participants or just memorization of the recording settings. To this goal, the token path is the further analysis of people with multiple recordings, where one recording has been used to train the target model, and the other has not. These cases are of particular interest, because the same face has been used in the target training but not the same recording. Due to this, any boost in the performance of the attacking pipeline for these cases would be indicative of the memory of facial information in the target model.

Our goal is to create an attack model on the previously designed eye-tracking [21, 33]. For training this target model (eye-tracking model), the model was initially pre-trained on the GazeCapture data set [33] which consists of approximately 1.5 million frames from 1450 participants with an 80%, 10%, 10% train, validation, and test split. The hyperparameters of the training are 35 epochs with batch size 16, weight decay 0.0001, momentum 0.9, global learning rate 0.0001 was used and decayed by a factor of 10 every five epochs. The features for the CNN are left and right eye crops in addition to face crop and the grid location of the face (Fig. 1). Then, using the pre-trained model weights (transfer-learning), the model was trained [21] on our separate data set with the same hyperparameters. The private recordings of participants with single recording were randomly divided into training, validation, and test sets. The remaining recordings related to participants with multiple recordings were randomly divided into the training, validation, and test set while keeping different recordings of the same individuals in different sets. The numbers of recordings in each set can be seen in Table 4. Note that we have all the information (the recordings, their labels, and if they are used in training of the target and which frames were used in the training and if they are from individuals with other recordings available) about the training set of the target at this point in the study. In our attack model, recordings of participants with a single recording were randomly divided into three separate sets: training, validation, and test sets. There were 54 participants with multiple recordings, where they have at least one recording inside the training set of the target model and at least one recording not in the training set of the target model. The recordings from these 54 participants were divided into in_training (those recordings used in the target model’s training set) and out_training (those recordings not in the target model’s training set). The in_training recordings were all put in the attack model training set as well. The out_training recordings were randomly divided into training, validation, and test sets for the attack model (Table 4).

**Table 4:** Data distribution

	Train	Valid	Test
Number of records			
Total	242	170	198
*Y*_instance_ = 1^a^	140	73	93
*Y*_person_ = 1^b^	159	87	114
Number of frames			
Total	83477	66755	73857
*Y*_instance_ = 1	46515	30072	35999
*Y*_person_ = 1	50654	32950	41456

^a^The labels are set to 1 if the recording was used in the target network’s training.

^b^The labels are set to 1 if at least one recording of the person was used in the target network’s training

For the labeling of the attack model(s), (*Y*), two were produced. In $Y_{\text{instance}}$, the labels were set to (1) for all the frames if the recording was in the target network’s training set and (0) otherwise. In $Y_{\text{person}}$, labels were set to 1 if at least one recording of the person was used to train the target network. In other words if there are two recordings of person *A*, *A*1 and *A*2, then if *A*1 was used in the training of target network but not *A*2, then $Y_{\text{instance}}(A1)=1$, $Y_{\text{instance}}(A2)=0$, but $Y_{\text{person}}(A1)=1$ and $Y_{\text{person}}(A2)=1$.

#### Classification pipeline

There are two classification pipelines used as attacks on the target model. The first one labels the recording based on whether the recording has been in the training data or not (instance). The second one labels the recording based on whether the participant has been in the training data or not (person). They both have very similar architectures, different only in the last step with different labels. The pipeline can be divided into three parts, parameters collection, classifier/frame labeling, and record membership inference.

##### Parameter collection

The original trained eye-tracking model (for more on the model, one can refer to Haque et al. [21]) was used to derive not only the activations, output, and label but also the gradients and loss for each frame in each recording. This was performed by feeding the trained network the frame and label and extracting the calculated parameters (with different dimensions (*x*)).

##### Classifier/frame labeling

This part can be viewed as a two-step section, encoding and frame classifying. In the encoding part, for any frame, the parameters from the previous step were fed to a separate fully connected network [dimension (*x*, 128) with dropout 0.2], with one hidden layer [dimension (128, 64)], so that the information will be encoded with specific encoders for each parameter type. For each input, they would be encoded to a 64 dimension. Then, the outputs of the encoding parts will be fed to another fully connected network with three hidden layers [(64+$\cdots$+64, 256), (256, 128), (128, 64), (64, 1), followed by a Sigmoid function instead of ReLU at last step], to train for classification using the encoded information (similar to the work by Nasr et al. [8]). The output of this second part is a number between 1 and 0 which is the probability the model assigns to the frame being in the training set for the original eye-tracking model or not. The rectified linear unit (ReLU) was chosen as the activation function on all layers except the final one, which was chosen to be a Sigmoid function, to produce the output probabilities. Binary cross-entropy was used as the loss function for the training of the labeling network. The training hyperparameters for this part are 100 epochs with batch size 16, weight decay 0.0001, momentum 0.09 and learning rate of 0.0005.

##### Record membership inference

The outputs of the classifier part are for each frame. However, any recording either has been part of the target model training or not. In this step, the labels of all the frames from each recording (the number of frames is different for different recordings) are used to produce a final membership inference for each recording. Here different moments (mean, variance, skewness, and kurtosis) and the entropy have been captured for each recording to train the SVM from the sklearn library from python with linear kernel and tolerance for stopping $10^{-3}$ to label each record. In short, SVM uses parameters of labels of all frames gathered from each recording to label that recording.

#### Patient membership inference

The infrastructure of “Classification pipeline” is utilized here to give labels not based only on the recordings being in the training or not (“Record membership inference”), but based on participant/patient being part of the training set or not. This is tackled by relying on specific participants who had more than one recordings. In these cases, the focus is on the analysis of those where one recording of a participant is in the training of the target model and another recording of the same participant is not in the training of the target model.

### Experiments

All the sections in “Classification pipeline” have been applied in two experiments. The Instance model (trained on the data set with Instance label); and the Person model (trained on data set with Person label). Fifty-four records have different Instance labels and Person labels. These records have been assigned to the training, validation, and test sets.

To ascertain if the recorded data include facial specifications of the participants recorded in the network, or properties of the specific frame used in the training set (the second aspect of methodology), we analyzed the images from participants with more than one recording. Table 3 shows that from 54 of such recordings, 19 had been used in the attack model’s training set, while 14 were in the validation set and 21 in the test set. These are the recordings that are not used in the training of the target model directly but are from the people who have other recordings present in that training set. Suppose the facial features of participants recorded in the network are making the predictions of the first part possible. In that case, they should show their effectiveness in the “Person” model (model trained by Person *Y* labels) in labeling these participants, because they are from the same people but only different recordings.

Different sets of parameters collected in the parameter collection step of the classification pipeline (“Parameter collection”) were used to determine which ones provided helpful information to improve the model results in the frame labeling step of the classification pipeline (“Classifier/frame labeling”). The loss (binary cross-entropy) on the validation data set for several sets of parameters can be seen in Table 1. The first set of parameters include the output of the two last layers of the target model (FC2 and FC1 in Fig. 1). The second set, in addition to parameters from the first set, includes the two last gradients before the last gradient of the target model. The third parameter set contains three additional gradients in comparison to the second parameter set (FC-FG2, FC-F2 FC-E1 also added, Fig. 1). The fourth set of parameters contains parameters of the second set in addition to loss and label from the target network. The fifth parameter set contains three gradients instead of two of the set four. While adding more parameters provides more data from the target model, this inspection of different sets of parameters is useful for understanding the contribution of different parameters from different layers of the target neural network. The model with two outputs, three gradients, and loss (from target model) and label as input was selected for the rest of the work, because it has the lowest loss.

## Data Availability

All patient data used in this study involves full face images, and is protected under HIPAA for meaningful release. The eye-tracking algorithm has been commercially licensed and is available upon request through Emory’s Technology Licensing Office.

## Abbreviations

ML: Machine learning
AI: Artificial intelligence
PHI: Protected health information
HIPAA: Health insurance portability and accountability
CNN: Convolutional neural network
FC: Fully connected
ROC: Receiver operating characteristic
AUROC: trapezoidal area under the receiver operating characteristic curve
PR: Precision–recall
AP: Average precision
ReLU: Rectified linear unit
SVM: Support vector machine
DP: Differential privacy
